# Localized Bullous Pemphigoid Triggered by Surgical Trauma: A Case Highlighting the Koebner Phenomenon

**DOI:** 10.7759/cureus.104408

**Published:** 2026-02-27

**Authors:** Marwa Faik Ouahab, Soumiya Chiheb, Awatef Kelati

**Affiliations:** 1 Dermatology Unit, Cheikh Khalifa International University Hospital, Mohammed VI University of Health Sciences, Casablanca, MAR

**Keywords:** autoimmune blistering diseases, bullous pemphigoid (bp), clinical dermatology, koebner phenomenon, surgical trauma

## Abstract

Bullous pemphigoid (BP) is the most common autoimmune blistering disease in the elderly. Although usually spontaneous, it may be triggered by skin trauma. We report the case of a 90-year-old woman who developed localized BP along a surgical scar one month after orthopedic surgery. Lesions later spread to the trunk. Histology revealed a subepidermal blister with eosinophils, and immunofluorescence confirmed C3 deposition in addition to circulating anti-BP180 antibodies. Clinical improvement was observed following the initiation of topical corticosteroid therapy.

## Introduction

Bullous pemphigoid (BP) is the most common autoimmune blistering disorder [1], typically affecting elderly patients [2]. It classically presents with tense blisters on urticarial plaques, primarily located on the trunk and extremities, with intense pruritus [3]. Histopathological examination reveals a subepidermal detachment with the presence of eosinophils, while direct and indirect immunofluorescence shows IgG and/or C3 deposition, along with circulating autoantibodies targeting BP180 and BP230 [3]. Although BP often presents spontaneously, mechanical trauma, surgical interventions, or radiation have been described as potential triggering factors [4]. The Koebner phenomenon, classically associated with psoriasis, vitiligo, and lichen planus [5], refers to the appearance of lesions in areas of cutaneous trauma, and has also been reported in BP [6]. We present a case of localized BP manifesting at the site of a surgical scar, highlighting the role of the Koebner phenomenon in disease expression.

## Case presentation

A 90-year-old woman with a history of well-controlled hypertension treated with perindopril 5 mg once daily and acetylsalicylic acid 75 mg once daily, underwent open reduction and internal fixation for a right distal femoral fracture. The postoperative course was uneventful. A review of perioperative and postoperative medications revealed no newly introduced drugs.

**Figure 1:**
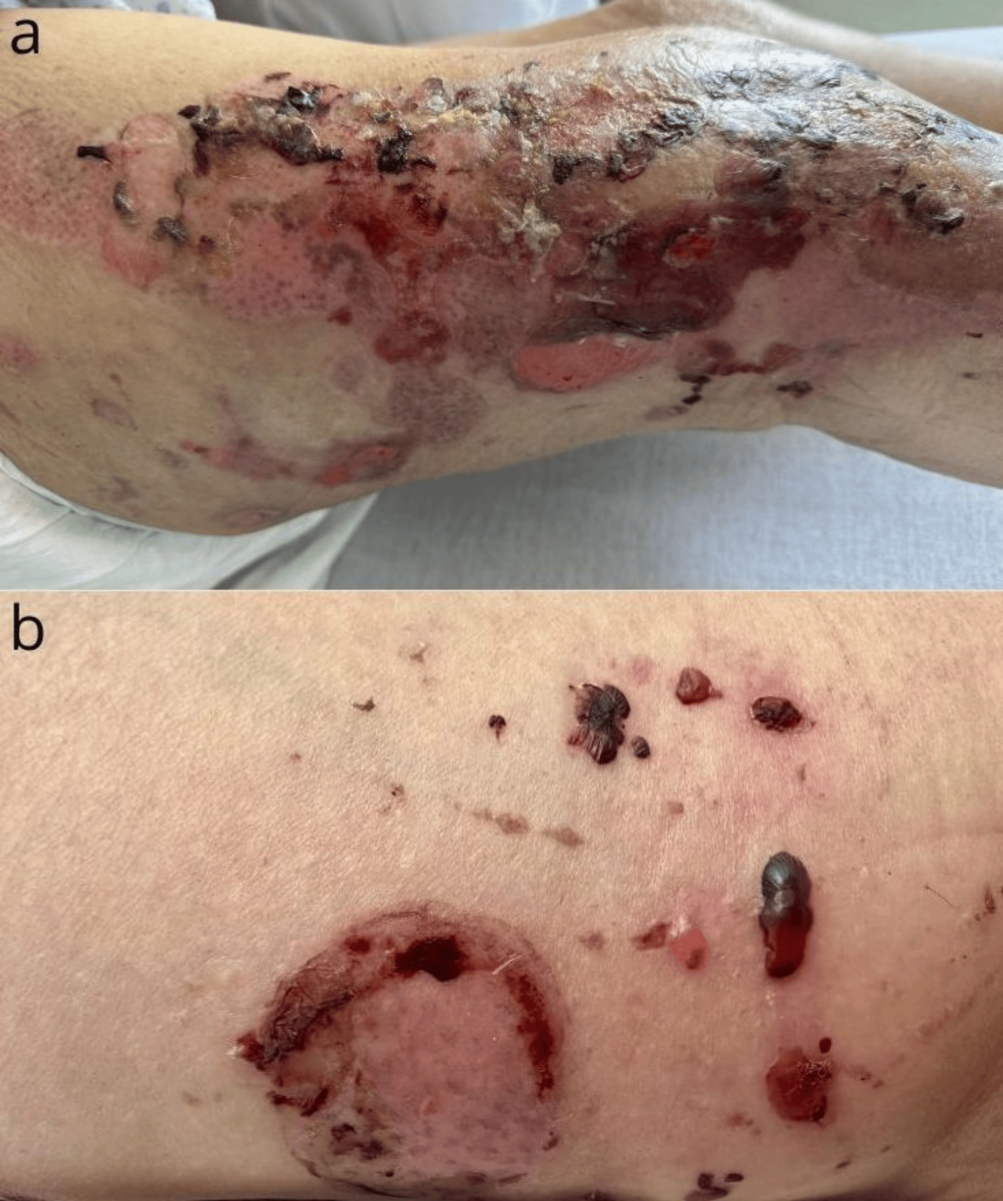
Multiple post-bullous erosions overlying erythematous skin, localized to the surgical site of the femoral fracture (a), with tense hemorrhagic blisters on the right thigh (b).

**Figure 2:**
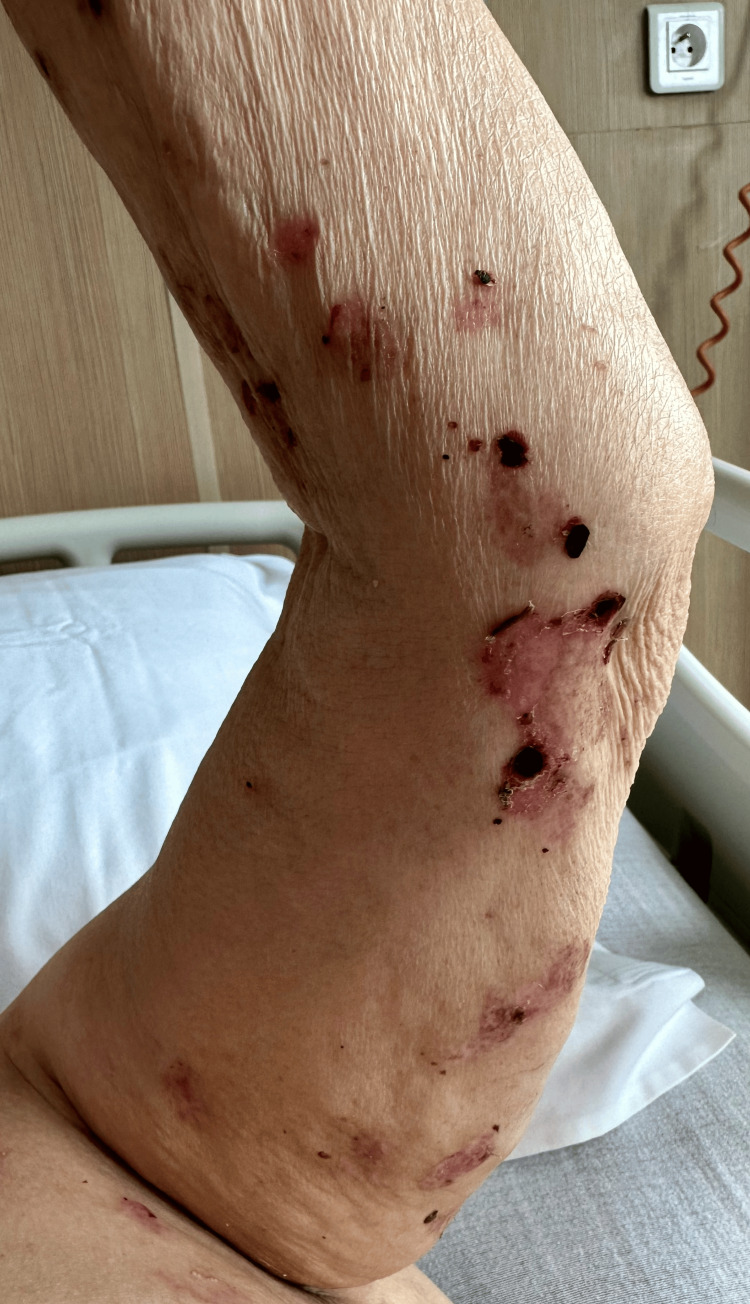
Extension of the initial bullous lesions onto the arms.

One month after surgery, she developed multiple tense bullae on pruritic urticarial skin, both clear and hemorrhagic, localized along the surgical site (Figure 1). Two weeks later, new lesions appeared on the arms (Figure 2). No mucosal involvement was observed. Several differential diagnoses were considered, including bullous drug eruption, bullous cellulitis, friction blisters, and bullous pemphigoid.

**Figure 3:**
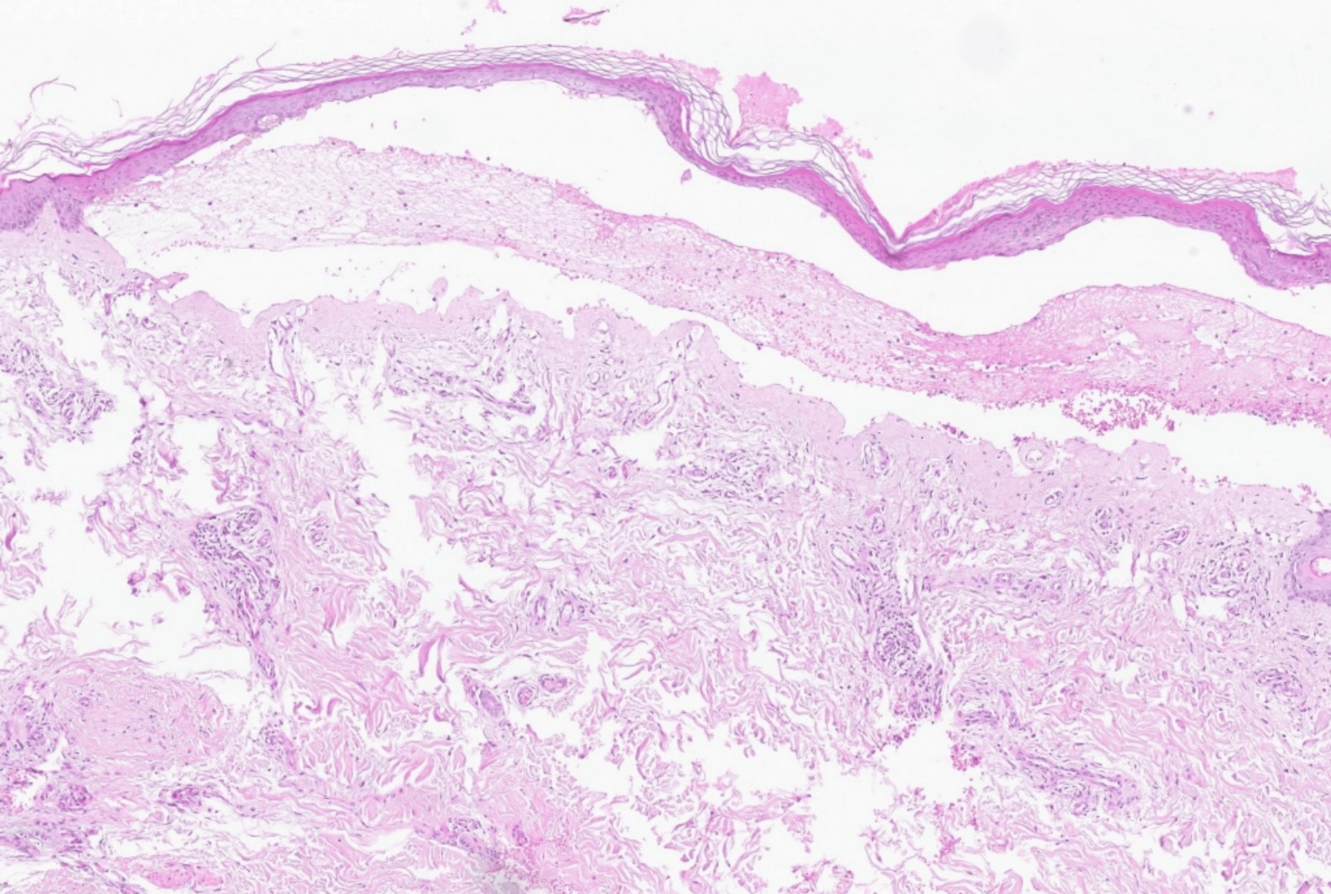
Histopathological examination of a bullous lesion showing subepidermal blister with eosinophils and mild superficial perivascular lymphohistiocytic infiltrate (H&E staining, low magnification; original magnification ×40). H&E: hematoxylin and eosin

**Figure 4:**
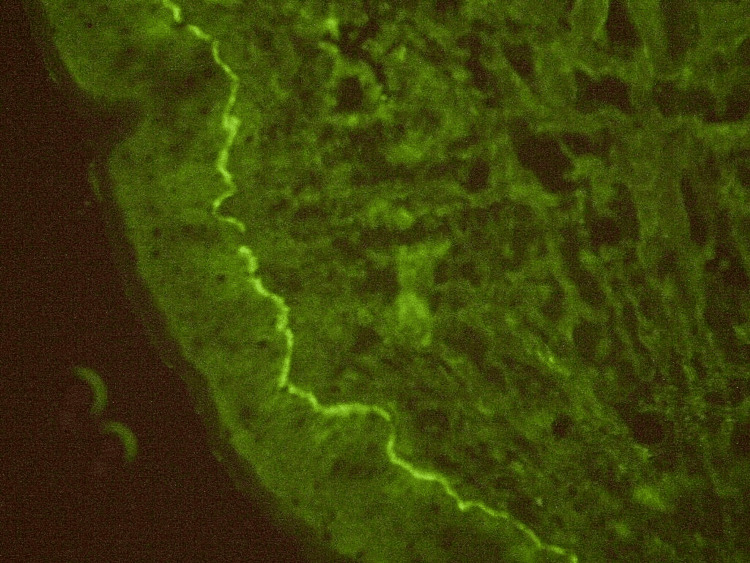
Direct immunofluorescence showing linear deposition of C3 along the dermoepidermal junction.

A skin biopsy revealed a subepidermal blister with a dense eosinophilic infiltrate (Figure 3). Direct immunofluorescence demonstrated linear deposition of C3 along the dermoepidermal junction (Figure 4). Laboratory evaluation showed positive anti-BP180 IgG autoantibodies (32 U/mL, reference <20 U/mL) and significant eosinophilia (1,5 g/L). Based on clinical and paraclinical findings, bullous pemphigoid was diagnosed.

**Figure 5:**
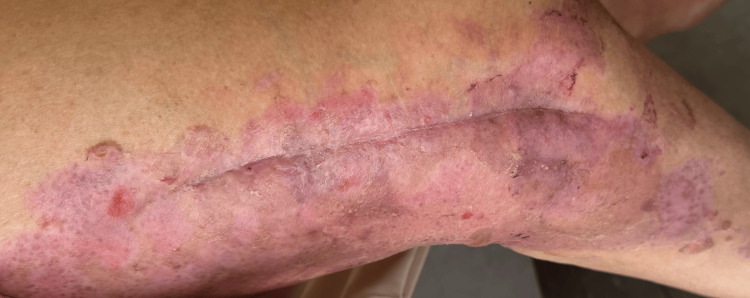
Clinical aspect at one month follow-up showing complete resolution of initial bullous lesions. Note improved definition of the surgical scar after resolution of the skin lesions.

The patient was treated with high-potency topical corticosteroids once daily, resulting in complete resolution of lesions within one month (Figure 5).

## Discussion

Localized bullous pemphigoid (BP) is an uncommon clinical variant that primarily affects the lower limbs [7]. In some cases, localized lesions may precede the development of generalized disease by weeks or even months, as seen in our patient [8].

In this case, the initial distribution of lesions along the surgical scar suggested an isomorphic response consistent with the Koebner phenomenon, a process whereby trauma or skin injury induces lesion formation in predisposed individuals. Koebnerization has been documented in several autoimmune blistering disorders, including BP [9]. Local trauma is a recognized trigger, with reports of koebnerization following various insults such as surgical wounds, burns, and phototherapy [9]. Specifically, trauma may facilitate blister formation by disrupting the basement membrane zone and exposing normally sequestered antigens, thereby initiating an autoimmune response [8].

The current leading hypothesis for trauma-induced koebnerization in bullous diseases involves epitope spreading. Epidermal injury and dermal inflammation are thought to unmask antigens like BP180 in genetically or immunologically predisposed individuals, promoting new lesion development [10]. 

Antibodies against BP180, a transmembrane component of hemidesmosomes, are central to BP pathogenesis. Although BP230 is intracellular and less accessible to autoantibodies, it may be secondarily targeted through this spreading mechanism, contributing to disease propagation [11].

Additional factors may explain the localized presentation in our case. Postoperative edema following orthopedic surgery could have increased local concentrations of circulating BP180 and BP230 autoantibodies, known to exist in low titers in up to 7% of healthy individuals [12], as well as immune mediators and effector cells, thereby favoring lesion formation in that region [13]. This mechanism aligns with other reports of unilateral lower limb BP associated with chronic edema or venous stasis, highlighting the role of vascular compromise in localized disease [14].

In the absence of other known triggers such as radiation, burns, or neurological impairment, this case reinforces the concept that localized trauma, inflammation, and edema may have contributed to inducing BP via koebnerization, particularly in anatomically vulnerable regions [15].

## Conclusions

This case highlights the potential role of localized trauma, postoperative inflammation, and tissue edema in the development of bullous pemphigoid lesions. The initial localization of bullae to the surgical site, followed by secondary spread, is suggestive of Koebnerization as a contributing factor in disease pathogenesis. Awareness of this phenomenon is important for clinicians, as bullous pemphigoid should be considered in the differential diagnosis of postoperative blistering eruptions, allowing for early diagnosis and appropriate management.
